# April

**Published:** 1890-04-12

**Authors:** 


### April.

The whole face of nature is now turning from the iron clasp of winter to kiss the balmy lips of returning spring, and to welcome the bridegroom sun. The storms of winter have nearly passed, and one unbounded spring will soon encircle all. It is true that April showers will fall, and that at times, especially in the earlier part of the month, we may be annoyed by a temporary return of the blustering winds and chilling rains of March, but surly winter has passed off for the north, and with him reluctantly follow his "ruffian blasts." Still it is not wise altogether to discard winter clothing, although we may be strongly tempted to do so by the warm and sunny days which generally occur. The temperature, however, is yet too uncertain for mortals to be incautious, as snow storms and hail storms are not infrequent even in April. Men and April have been compared. For "Men are April when they woo!" The time has not yet arrived for those who went abroad for the winter to return to the British Isles. After the 15th of April, formerly called "swallow day," from swallows, "delightful messengers of spring" being first generally seen in England, people abroad may begin to think of returning, with some expectation of having at least tolerable weather. The term April or Aprilis is presumed to be derived from the Latin *Aperio* to open, and the month was represented as a young girl clothed in green, with garlands of myrtle and hawthorn buds, holding in one hand primroses and violets, and in the other the zodiacal sign Taurus, or the Bull, into which constellation the sun enters during this month. Among the Romans April was sacred to Venus, as being the period when united energy bursts forth into new life. Or as old Chaucer had it in 1328:

"Whanne that April with his showers sote,
The draughte of March hath parced ye rote,
And bathed every veine in swiche licure,
Of whiche virtue engendered is the flour."

On the 1st of April, formerly known as "All fools day," it was the custom for people to endeavour to fool their neighbours by sending them on "sleeveless" errands, &c., The origin of "All fools day" seems shrouded in mystery, but a similar epoch was observed by the Romans, although owing to alterations of the kalender it was not this identical anniversary. Some have surmised that All fools day, is attributable to the mistake of Noah in sending the dove out of the ark before the waters had subsided. Others have opined that the custom of imposing upon, and ridiculing people on the 1st of April may have an allusion to the mockery of our Saviour by the Jews. Happily, however the custom originated, it is now more honoured by the breach than by the observance. But in former times many placidly submitted to the custom of being made fools on the 1st of April, who would have bitterly resented any attempt to play them off in a similar manner on any other date. As a specimen of what was formerly received as April fool day wit, we note the following. A beau of the period is passing jauntily along, when an urchin shouts "Sir! there's something out of your pocket!" "Ah! what! where?" says the beau. "Your hand, Sir," replies the urchin. "Ah! you April fool!" which is received without offence by the beau. April 2nd was once signalised as the day of St. Francis. This personage appears to have been in his early career a poor representative of the Eastern Buddha, commencing his life by abstinence and meditation. But afterwards he essayed conjuring tricks which Buddha would have scorned, and Maskelyne and Cooke despised. He held burning coals in his hands without injury; he sat in a hot oven; he floated on water in his cloak instead of in a boat; and he defied a whole party of ghostly devils, notwithstanding a grievous beating from the satanic hands! April 2nd, is also the anniversary of Lord Nelson's victory in 1801, at Copenhagen. Then eighteen sail of the line of the enemy's fleet were either captured or destroyed. We wonder what will be the fate of our new ships—if ever they are built—should another Copenhagen be fought. We recollect the time when it was gravely advised in the *Times* that country gentlemen should walk about with acorns in their pockets, which they should distribute in hedge-rows so that oak trees might be forthcoming for future men-o-war ships. This is, however, as regards ships, the iron age, and it may be doubted if as good an account of the enemy be rendered, as when wooden vessels were opposed to wooden vessels.

April 6th, Easter day, is observed all over Christendom with peculiar rites. And so it was long before the Christian era, as we are told in Ramsay's "Travels of Cyrus." The period was noted among eastern nations—among the Persians especially—for the triumph of the sun of Nature, as with the Christians for the triumph of the Sun of Justice, the Saviour of the world, over death by his Resurrection. There are also some peculiar Easter customs formerly much more prevalent than they are now. One is "lifting" or "heaving." On Easter Monday it was the custom for the men to heave the women, and on Easter Tuesday for the women to heave the men. The writer can recollect that in the part of England he then resided, it was rather a risk for a man to go abroad before twelve o'clock on an Easter Tuesday, as he was very likely to be seized by a number of athletic females, "heaved," perhaps kissed, and made to pay his "footail," or fine! An old writer said the custom of heaving had been handed down from the bewildering ceremonies of the Romish Church, as an absurd performance of the Resurrection! Easter Monday and Easter Tuesday have long been regarded as holydays to which the once famous Greenwich fair bears testimony. And in these days we have Bank holyday on the 7th. As illustrating change of manners and customs, it may be mentioned that so late as April 6th, 1803, a Colonel Montgomery and a Captain Macnamara fought a duel at Primrose Hill. The former was shot dead on the spot, and the latter was tried at the Old Bailey. He was acquitted by a jury of his countrymen as a "man of honour!" On April 9th, 1483, the great Lord Bacon died. It was said of him that although no admirer of money he had the unhappiness to be defiled therewith. "His connivance of the bribery of his servants made them his masters, and wrought his ruin." April 16th is sacred to the memory of the venerable benedict, Joseph Labre, who died in the odour of sanctity in 1783. But, says Hone, if such a man as the venerable Joseph can be called a man he was one of the silliest that ever lived; and one of the dirtiest that ever died in the odour of scantity. His career was very much that of an Indian fakeer. He lived in a hole, endured much privation, scourged himself, and became a prey to insects which the term "pediculus" describes most accurately. We don't bribe, we don't fight duels, and we don't saint men who are the prey of the pediculus in these happy days! On April 18th, 1689, Judge Jeffries died after hanging upwards of 600 people, and we have a reminder of the Judge in the various lanes still called "gibbet" in different parts of the country. On April 18th also, 1663, the first play-bill was issued by the company then acting at Drury Lane. We wonder if they charged for it? Now-a-days, we are, at most theatres, irritated by an extortionate charge for a programme. This, we have reason to know, deters many of irritable temperament and disposition visiting such theatres.

On April 20th the sun enters Taurus, and an old writer said, "there is with the incoming of spring an out-going from town." But the present generation do not go out of town on the 20th of April. However much in these days we may love the country at this season of expanding buds, men of the present day are like Horace. They admire rural life, but for all that they remain pretty much the modern representative of Augustus. April 23rd is St. George's day. St. George is the patron saint of England. But who Saint George was no one knows. Nevertheless, St. George "the blyssed and holy martyr is patron of this realm of England." And although we do not know who he was, are not his deeds chronicled, especially as regards his fight with the fiery dragon, in that delicious book of our childhood, "The Seven Champions of Christendom?" St. George was the ancient British war-cry, and the legend is, that it has struck terror into many an enemy's heart. We believe, however, that the yell Tommy Atkins now makes when he charges, is infinitely more terrific than any ancient cry of St. George could possibly be! April 25th belongs to St. Anianus, whilom a cobbler, of Alexandria. St. Mark having burst his sandal, applied to the cobbler to get it mended. As payment St. Mark taught Anianus Christianity, and Anianus taught other people, becoming eventually the first Pontiff of Alexandria. Even in the present day in the East, persons rise from obscurity to prominence, much more frequently than they do in the more prosaic West. April 30th is the day of St. Catherine of Sienna, Catherine was a sort of female fakeer (and by-the-bye a female fakeer is never seen in the far East). Catherine lived on herbs like a female Nebuchadnezzar, took no sleep, wore a skin of iron with points or nails inside, flogged herself till the blood ran, never spoke for three years, and notwithstanding all this often saw the devil, which appears rather hard on Catherine! But she must have been still more disgusted, when an old woman, whom Catherine essayed to cure of the cancer and failed, declared publicly that Catherine was not so good as she should be!

We should not omit to mention that about the middle of the month the lark is first heard, as some one more poetically than practically observed, "being married to morning by a sweet hymn." About the same time also the nightingale begins to sing the "lulling lay." Cowper's poem of the nightingale singing with a thorn in its breast may be recollected. Well, a great many of us have to sing, or at least look cheerful with metaphorical thorns in our breasts. We all recollect the story of the clown who convulsed his audience with laughter, while his wife and children were dying in their lodgings. "Man is born to trouble, as the sparks fly upward," wrote patient Job some thousands of years ago. But there are two sorts of people who take their rue with a difference. Let us hope that the majority belong to that class who still fancy this earth fit for the home of the gods, and Pandora's casket yet unloosed.

